# Relapse/Refractory Paediatric B-ALL Case with CD19^−^ Phenotype Switching Indicating the Importance of Appropriate Diagnostic Approach and Targeted Treatment Adjustment—Case Report

**DOI:** 10.3390/ijms241713322

**Published:** 2023-08-28

**Authors:** Anna Prażmo, Patryk Jawoszek, Borys Styka, Monika Lejman, Agnieszka Zaucha-Prażmo

**Affiliations:** 1 Department of Pediatric Hematology, Oncology and Transplantology, Medical University of Lublin, 20-059 Lublin, Poland; agnieszkazauchaprazmo@umlub.pl; 2 Doctoral School at Medical University of Lublin, 20-059 Lublin, Poland; patryk.jawoszek@gmail.com; 3 Independent Laboratory of Genetic Diagnostics, Medical University of Lublin, 20-059 Lublin, Poland; borys.styka@wp.pl (B.S.); monikalejman@umlub.pl (M.L.)

**Keywords:** CD19-negative paediatric B-ALL, relapse/refractory, microarrays, blinatumomab, CAR T-cell

## Abstract

The case reported presents a rare CD19^−^ phenotype shift of an acute lymphoblastic leukaemia clone during relapse/refractory ALL in a paediatric patient. We explore possible reasons for the promotion of CD19-negative cell selection, including discrete mutations and anti-CD19 treatment, which is gaining importance as targeted therapies such as blinatumomab enter standard treatment protocols. A 9-year-old male patient was diagnosed with B lymphocyte acute lymphoblastic leukaemia. Initial standard genetic analysis did not show significant chromosomal aberrations, and the patient underwent chemotherapy in line with the intermediate-risk protocol. After initially achieving remission, the disease relapsed, and the patient required hematopoietic stem cell transplantation (HSCT). In-depth retrospective microarray analysis performed at this point revealed additional risk factors, particularly a loss of function *TP53 V173L* mutation. A second recurrence was diagnosed which prompted targeted treatment application (blinatumomab) and subsequent HSCT. The third leukemic relapse, diagnosed shortly after the second HSCT, limited treatment options to last-resort CAR T-cell therapy in Germany. Subsequent immunophenotyping revealed insufficient CD19 expression by ALL clones and disqualified the patient from treatment. The patient died in October 2019 from disease progression. The case highlights the importance of in-depth molecular diagnostics and monitoring of relapse/recurrent ALL cases to identify and manage risk factors during treatment.

## 1. Introduction

Advances in immunophenotyping and genetic screening techniques have contributed to a shift in the understanding of acute lymphoblastic leukaemia (ALL) as a heterogenous group of diseases defined as malignancies of the lymphoid line of white blood cells (WBCs) characterised by a rapid development of large numbers of immature lymphocytes. On a molecular level however, multiple different heritable and acquired mutations of genes involved in lymphoid cell proliferation can lead to either dysregulating the cell cycle or accumulating subsequent harmful mutations [1]. Many of these mutations have been described and linked to disease development, such as *C-MYC* translocation or *ETV6::RUNX1* and *BCR::ABL1* fusion genes, but some are less common and often include numerical mutations, such as hyperdiploidy. Different combinations of these changes are reflected in ALL clone phenotypes through the expression of different antigens and receptors [2].

Multiple pieces of evidence suggest that de novo mutations in tumour progenitor cells may lead to a phenotypic shift during treatment or in the case of a relapse [3]. As a treatment escape process, changes in a phenotype often lead to an emergence of a less-differentiated clone and can involve myeloid lineage switching [4]. Determining the unique immunophenotype is important not only for diagnostic purposes, but also treatment purposes. While chemotherapy remains the first line of treatment for ALL, multiple targeted therapies involving enzyme inhibitors and immunomodulation, such as blinatumomab and CAR T-cells, rely solely on identification of specific cell surface markers. Targeted therapies are often used as a supportive treatment to haematopoietic stem cell transplantation (HSCT) for relapsed ALL, showing a significant increase in overall survival rates [5]. Even though the predictive value of specific antigenic combinations on treatment outcome remains to be assessed for novel methods such as CAR T-cell, key antigens must be expressed to induce response based on treatment mechanism. The presence of such targets must be reassessed, possibly during the treatment cycle and always in the case of a relapse.

This case report aims to demonstrate, through an example of a relapse/refractory paediatric B-ALL case, the need for in-depth genetic analysis using microarrays and immunophenotyping at multiple points during treatment, to ensure the most effective approach and realistic prognosis. We describe a rare case of a CD19-negative B-ALL phenotype acquired over the course of treatment and multiple relapses in a 9-year-old patient, which disqualified him from CAR T-cell therapy, eventually leading to his death. Based on available literature, we try to assess whether additional risk factors could be inferred from molecular analysis, and how to effectively monitor a recurrent disease for phenotype changes. This is particularly important in 2023, as the presented anti-CD19 treatment methods are now significantly more available through standard treatment protocols, which might increase the incidence of resistant cases, thereby requiring close monitoring.

## 2. Case Presentation

**Figure 1:**
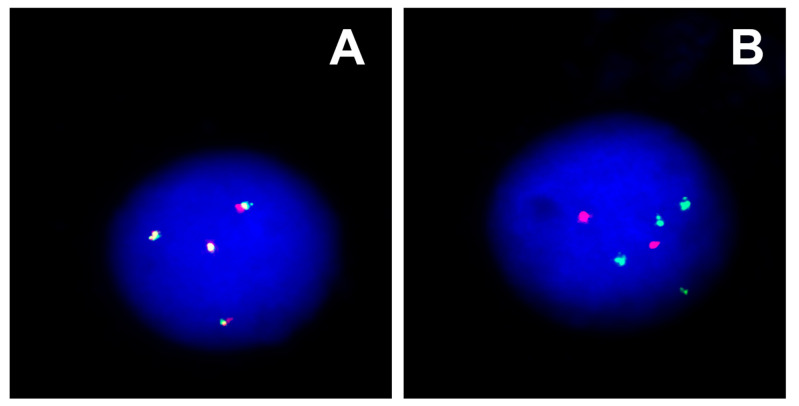
FISH analysis showing additional gene signals: (**A**) four copies of KMT2A (red-green) and (**B**) four copies of BCR (green) and two copies of ABL (red).

**Figure 2:**
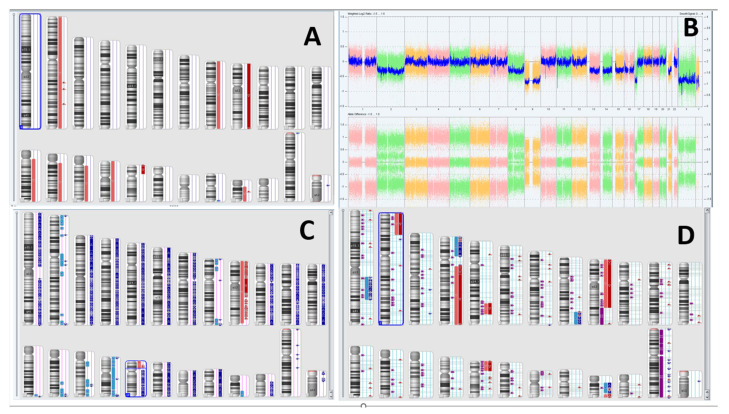
(**A**) Normal karyotype seen at diagnosis. (**B**) Microarray suggesting first evidence of hyperdiploidy. (**C**) Further karyotype generated using Normal Diploid overlay—duplication of most chromosomes is evident, with a noticeable LOH at short arm of chromosome 17. (**D**) Karyotype analysis at first relapse. Own work using Chromosome Analysis Suite, 2017 and Affymetrix GeneChip 2.7 HD.

In October 2015, a 9-year-old male was admitted to the Department of Paediatric Haematology, Oncology and Transplantology of the University Children Hospital in Lublin. The patient was referred to the hospital by a GP after the CBC results suggested a malignant disease. The boy presented with tiredness, anaemia, neutropenia, and hepatosplenomegaly. Peripheral blood smear and myelogram revealed 43% and 93% blasts, respectively. The patient was diagnosed with B-ALL. Immunophenotyping showed expression of CD10 (95%), CD19 (96%), CD22 (98%) and CD79a (96%). Cytogenetic analysis revealed a normal karyotype with no structural or numerical aberrations of chromosomes. There was no evidence of *BCR::ABL1*, *ETV6::RUNX1* fusion genes or *KMT2A* and *TCF3* rearrangements. However, additional signals were observed from molecular probes complementary with *ETV6*, *RUNX1*, and *TCF3,* which suggested a hyperdiploidy, despite a seemingly normal karyotyping result (Figure 1). Retrospectively, and outside the standard treatment protocol, microarrays were performed using the Affymetrix GeneChip 2.7 HD, which provided further evidence of a hyperdiploid karyotype (Figure 2). The patient was classified to an intermediate risk group based on his age (<9 years old) and initial response to treatment. The peripheral blast count on the 8th day of chemotherapy was <1000/µL, and the percentage of blasts in myelogram at 15 days of treatment was <2%. Intensive chemotherapy according to the ALL-IC-BMF 2009 protocol [6] concluded in July 2016.

In January 2017, a very early isolated BM relapse was diagnosed, and a subsequent genetic analysis of somatic karyotype and microarrays revealed previously non-existent karyotype changes: 46,XY,t(9;17)(p10;p10). There was evidence of complex gain/loss events as well as duplication of several autosomes including chromosomes 1, 2, 4, 5, 8, 9, and 21, and deletion of the short arm of chromosome 17 with a loss of heterozygosity (LOH) (Figure 2). Additional genetic tests confirmed a pathogenic loss of function mutation *TP53 V173L*. Immunophenotyping profile presented CD10 (95%), CD19 (95%), CD22 (95%) and CD79a (97%). Based on the IntReALL definition of standard and higher risk strategy group classification, the patient was classified to the high-risk (HR) group due to very early relapse [7]. According to IntReALL, all HR patients are eligible for allogeneic hematopoietic stem-cell transplantation after achieving a complete second remission. Chemotherapy for HR patients was introduced, and after achieving a complete remission, HSCT from the patient’s sister was performed. The post-transplant period was uncomplicated, and the patient presented with full donor chimerism.

A second recurrence occurred in February 2018. Karyotyping showed 46,XY//46,XX (68% XY/32% XX), which was expected after allo-HSCT from the sister donor. Fluorescent in situ hybridisation (FISH) revealed no structural rearrangements; however, there was a signal missing from *ETV6* and an additional signal from *RUNX1*. Microarrays were not performed at this stage due to mixed chimerism (32% of donor cells). The immunophenotyping profile was similar to that in the first relapse, with CD10 (95%), CD19 (95%), CD22 (96%), and CD79a (97%). At this point, IntReALL HR 2010, version 2.0, HIA block [7] was introduced. Additionally, three courses of blinatumomab were administered between 13 April and 4 August 2018. The first cycle was complicated by fever and neurological symptoms, such as aphasia and hemiparesis. This required ceasing blinatumomab and implementing dexamethasone and mannitol treatment. After achieving improvement in the patient’s clinical state, therapy with reduced doses of blinatumomab was continued and was further well-tolerated. The patient received the next two cycles of blinatumomab, and additional tests showed unsuccessful treatment response. In September 2018, the patient underwent the second HSCT from the same family donor without post-transplant complications.

**Figure 3:**
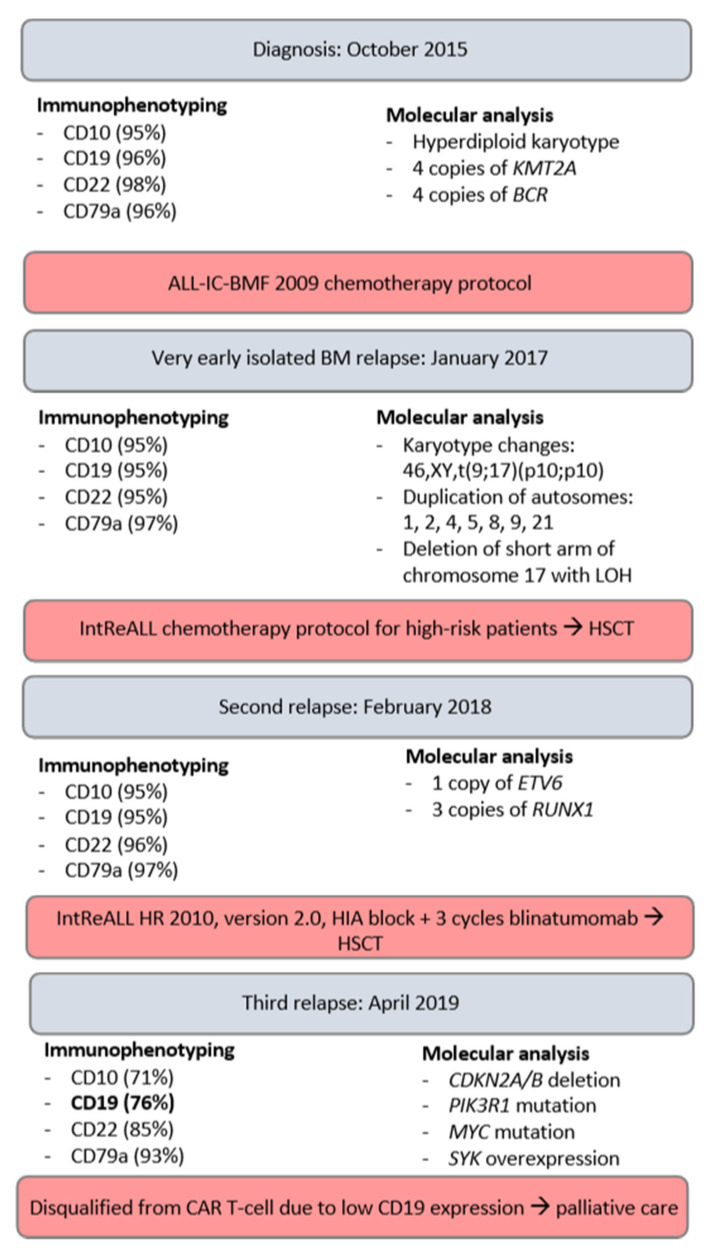
Overview of the diagnostic and treatment process, highlighting crucial parameters.

A follow-up bone marrow examination in April 2019 revealed 94% of blasts. Initial immunophenotyping in our clinic revealed changes in antigen expression: CD10 (71%), CD19 (76%), CD22 (85%), and CD79a (93%). After diagnosing a third leukemic recurrence, the patient was redirected for further testing and qualification for CAR T-cell therapy in Germany. He was also qualified for the INFORM (Individualized Therapy For Relapsed Malignancies in Childhood) registry based on inclusion criteria: relapsed ALL with >40% blasts in BM. Subsequent analysis in Germany concluded that CD19 expression was insufficient to qualify the patient for CAR T-cell therapy. Molecular analysis revealed *CDKN2A/B* deletion, *PIK3R1*, *MYC* mutation, and *SYK* overexpression. Based on CD22 expression, inotuzumab ozogamicin treatment was introduced in July 2019, but the patient did not respond to the first cycle (three doses). The second cycle induced a partial response with rising WBC counts. Due to treatment resistance and lack of subsequent effective solutions, therapy was ceased, and palliative care was introduced. The patient died in October 2019 due to disease progression. The overview of the described diagnostic and treatment process is presented in Figure 3.

## 3. Discussion

Despite good survival rates upon diagnosis of acute lymphoblastic leukaemia in children, relapsed/recurrent ALL is associated with a dismal prognosis and remains a leading cause of death from childhood cancer. Targeted therapies, including engineered T-cell therapies, are a new strategy that allows an increasing number of patients to establish durable complete remission [8]. Both blinatumomab and CAR T-cell therapy are based on an interaction of the drug with the CD19 target on malignant cells. Blinatumomab is a bispecific T-cell engager (BiTE), which enables the patient’s own T-cells to recognise and neutralise CD19^+^ cells, through combining two binding sites: the CD3 site for the T-cell and the CD19 site for the target cells [9]. Chimeric T-cell receptors are engineered receptor proteins that enable T-cells to target a specific antigen, in this case CD19. Expression of CD19 on ALL clone cells is a necessary provision to ensure treatment efficacy. The patient initially qualified both for blinatumomab and CAR T-cell therapy based on immunophenotyping at diagnosis and after relapses. The first treatment proved ineffective, while he was disqualified from the latter based on insufficient CD19 expression discovered in Germany.

As CD19 is a B-cell marker expressed in all stages of lineage development, CD19-negative B ALL is extremely rare, with only eight cases described in the literature [10]. This makes CD19 one of the key markers used in diagnostics and characterisation of B cell malignancies, with the WHO including CD19 as a lineage-defining marker for B-ALL. The early expression also contributes to CD19 being used in minimal residual disease (MRD) monitoring, and it has been suggested that lack of CD19 expression can lead to a delay in relapse diagnosis. Hence, Ghodke et al. suggest using at least four other B-cell markers (CD10, CD20, CD22, CD79a) in follow-up monitoring [10], which is routinely done in our clinic and has contributed to early diagnoses of relapses in our patient.

The exact mechanism of CD19^−^ relapse in our patient remains unknown. Initial presentation at diagnosis showed a normal karyotype, which did not suggest a higher relapse risk. We speculated that the karyotype could appear to be normal due to a highly heterogenic ALL clone population, with possible blood cell contamination. During diagnosis and testing, one sample collected from the patient must be divided into multiple specimens, creating a bottleneck effect, and preventing full spectrum analysis. Upon diagnosing a relapse, retrospective microarray analysis was performed from genetic material isolated at diagnosis which, together with FISH analysis, was sufficient to state that numerical mutations were present, resulting in a hyperdiploid karyotype. These aberrations are usually associated with a favourable prognosis; however, about 20% of patients relapse [11].

A more disturbing and potentially pathogenic mutation was undetectable using a standard karyotype and FISH analysis according to diagnostic protocol. Microarray analysis and Normal Diploid overlay revealed LOH at the short arm of chromosome 17, which raised concern about changes in the *TP53* gene. Subsequent INFORM analysis confirmed a loss of function *TP53* V173L mutation. This is a missense substitution mutation in exon 5 of *TP53*, which results in amino acid change p.Val173Leu. The change is located in a highly conserved *TP53* residue that is known to be functional, and, in silico, was shown to affect TP53 activity, possibly leading to increased malignancy. This mutation was previously described in patients with *TP53*-related disorders; it occurs in a region where several other missense mutations were described as being pathogenic for Li Fraumeni syndrome [12].

Loss of function mutations have been reported in connection with cases of CD19^−^ relapses after/during anti-CD19 treatments with CAR T-cell therapy, due to a dysfunctional or absent transmembrane domain of the CD19 surface antigen. These have been linked to the initial occurrence of relapse itself, being present in nearly all malignant cells [13]. A similar mechanism might have arisen as a result of selection during blinatumomab treatment for the third relapse, to which the patient showed poor response. A relatively small undetected CD19^−^ fraction could have dominated the tumour cell population after targeting CD19^+^ cells. Immunophenotyping performed in our clinic after administering three courses of blinatumomab presented decreased CD19 expression (Figure 3). This remains a hypothesis, as our clinic does not have access to the full immunophenotype profile and detailed genetic analyses performed in Germany after blinatumomab treatment.

Genetic analysis revealing potentially pathogenic mutations in key regulating suppressor genes such as *TP53*, as well as identifying cells with accumulated mutations, should raise additional concerns about a worse prognosis due to relapses and loss of surface antigens. The structure of the tumour cell population may change rapidly as a treatment evasion mechanism, and, therefore, we recommend repeating the analysis before implementing another drug with the same target as in a previous treatment. Disturbing evidence presented by Orlando et al. suggests that mutations resulting in CD19^−^ relapses could have been completely undetectable in samples collected as close as 1 month prior to clinical relapse [13], indicating further confirmation and measures are required to improve early diagnosis. This is especially important for patients belonging to high-risk groups, such those treated previously with a CD19-targeting protocol. It is important to note that, in 2016, blinatumomab treatment was not refundable, and had to be paid for by the patient’s family. This resulted in treatment delay, but also has other possible implications. As of 2023, blinatumomab is included in standard treatment protocols for relapsed ALL and is therefore much more widespread. Based on our experience with this patient, anti-CD19 treatment may induce pressure for CD19-negative clonal selection. This is consistent with the finding of Pillai et al. that CAR T-cell therapy is negatively affected by prior blinatumomab treatment, which increases the risk of CD19- MRD and relapse [14]. Hence, we could see an increase in recurrent CD19-negative ALL, resistant to last-resort treatments such as CAR T-cell therapy, thus increasing the need for close monitoring.

## 4. Conclusions

The case presented highlights the importance of in-depth genetic analysis using microarrays and close monitoring of ALL immunophenotypes as a prognostic factor for ALL treatment outcome and relapse risk. While standard diagnostic protocols provide evidence for the most common pathogenic mutation types, some of the changes can remain undetected. Microarrays are a useful diagnostic tool that we recommend using as early as at initial diagnosis, as they can help reveal discrete mutations, which can have a fundamental role in risk stratification and, therefore, the treatment approach.

## Data Availability

The data presented in this study are available on request from the corresponding author. The data are not publicly available due to patient confidentiality.
